# The relationship between red cell distribution width and all-cause and cause-specific mortality in a general population

**DOI:** 10.1038/s41598-019-52708-2

**Published:** 2019-11-07

**Authors:** Jingxue Pan, Yan Borné, Gunnar Engström

**Affiliations:** Cardiovascular Epidemiology Research Group, Department of Clinical Sciences, Lund University, Malmö, Sweden

**Keywords:** Biomarkers, Risk factors

## Abstract

Red Cell Distribution Width (RDW) could be a risk factor for developing various chronic diseases, and seems to be a prognostic marker in patients with cardiovascular disease (CVD) or cancer. Our aim was to explore the association between RDW and all-cause and cause-specific mortality in a general population. RDW was measured in 27,063 participants (aged 45–73 years) from the population-based Malmö Diet and Cancer cohort. After a follow-up of 19.8 ± 5.5 years, Cox proportional hazards regression analysis was used to study the relationship between RDW and all-cause and cause-specific mortality, with adjustment for confounding factors. A total of 9388 individuals (4715 men and 4673 women) died during the follow up. High RDW was significantly associated with all-cause mortality (HR, 4^th^ vs. 1^st^ quartile: 1.34, 95%CI: 1.24–1.45), cancer mortality (HR: 1.27, 95%CI: 1.12–1.44), CVD mortality (HR: 1.39, 95%CI: 1.21–1.59), and respiratory disease mortality (HR: 1.47, 95%CI: 1.06–2.03). The C-statistic increased significantly from 0.732 to 0.737 when adding RDW to a model adjusted for age and sex. There was a significant interaction between RDW and BMI with respect to all-cause mortality. We concluded that RDW is associated with mortality and propose that high RDW is a significant, but non-specific marker of mortality risk in the general population.

## Introduction

Red cell distribution width (RDW)^1^, a measure of anisocytosis, reflects the heterogeneity of the volumes of the red blood cells (RBC). RDW is often used clinically as a part of the diagnostic work-up in patients with anaemia. However, besides being a diagnostic tool in classification of anaemia, recent studies have shown that high RDW could be a risk factor for developing various chronic diseases and a prognostic marker in patients with established disease. In 2007, Felker *et al*.^2^ published the first study showing that the baseline value of this erythrocyte parameter may be useful for predicting both morbidity and mortality in heart failure patients. Since then, several studies^3–6^ but not all^7^ have reported relationships with incidence of various cardiovascular diseases (CVD). Some studies showed that RDW were related to occurrence of other chronic diseases, such as colorectal cancer^8^, chronic thromboembolic pulmonary hypertension^9^, and liver diseases^10^, and elevated RDW has been associated with all-cause mortality in community population^11,12^. Several mechanisms are believed to contribute to the relationships between elevated RDW and mortality, although the exact mechanism is unknown. For example, it has been hypothesized that high RDW could reflect inflammation, oxidative stress, anaemia, altered life-span or reduced deformability of the red cells, all of which could potentially increase the mortality risk. However, even though several studies have reported relationships between high RDW and mortality, few population-based studies have explored the relationships with deaths from different causes.

The aim of this study is to examine whether RDW is associated with all-cause mortality and different causes of death in a population-based study, the Malmö diet and cancer cohort (MDC), after controlling for potential confounders and anaemic related factors.

## Results

### Baseline characteristics

Participants with high RDW were more likely to be current and occasional smokers and high alcohol consumers, and had higher age, MCV, WBC, Apo A1 and vitamin B12 intake. There were inverse trends between RDW and BMI, systolic BP, use of lipid lowering drugs, use of BP lowering drugs, diabetes, Apo B and dietary intakes of iron and folate. Baseline characteristics of participants stratified by sex-specific quartiles of RDW were shown in Table 1.

**Table 1:** Baseline characteristics of individuals across sex-specific quartiles of red cell distribution width (RDW).

Sex-specific quartiles of RDW	Q1 (low)	Q2	Q3	Q4 (high)	Total	P value
Number of subjects	6709	6777	6743	6834	27063	
Number of death (%)	1767(26.3)	2117(31.2)	2428(36.0)	3076(45.0)	9388(34.7)	<0.001
RDW (fL)	36.78 ± 2.16	39.39 ± 0.59	41.43 ± 0.66	45.15 ± 2.74	40.71 ± 3.55	<0.001
Male Sex (%)	39.0	40.0	38.4	39.7	39.3	0.20
Age at screening (years)	56.85 ± 7.00	57.89 ± 7.45	58.63 ± 7.86	59.30 ± 7.98	58.17 ± 7.64	<0.001
BMI (Kg/m^2^)	26.19 ± 3.93	25.95 ± 3.92	25.65 ± 3.95	25.10 ± 3.95	25.72 ± 3.96	<0.001
Systolic BP (mmHg)	141.32 ± 19.58	141.09 ± 20.04	141.14 ± 20.21	141.18 ± 20.17	141.18 ± 20.00	0.92
MCV (Median (25%, 75%))	86.20(84.40, 88.40)	88.30(86.60,90.00)	90.10(88.20,92.10)	93.00(91.00, 95.00)	89.30 (86.80,92.00)	<0.001
Current and occasional smokers (%)	14.3	21.5	29.6	46.9	28.1	<0.001
Use of lipid lowering drugs (%)	3.4	2.8	3.2	3.0	3.1	0.31
High alcohol consumption (%)	3.1	3.5	4.2	6.4	4.3	<0.001
Use of BP lowering drugs (%)	18.7	18.0	17.4	17.7	18.0	0.21
Low physical activity (%)	25.4	24.3	24.3	26.3	25.1	0.02
Use of anti-diabetes drugs or Diabetes history (%)	4.7	3.2	2.0	2.0	3.0	<0.001
Low education level (%)	42.7	41.0	41.5	42.2	41.8	<0.001
Anaemia (%)	3.5	2.6	2.7	3.5	3.0	0.001
WBC (×10^9^/L)	6.06 ± 1.50	6.29 ± 1.66	6.45 ± 1.72	6.73 ± 1.90	6.39 ± 1.72	<0.001
Haemoglobin (g/L)	141.42 ± 13.80	141.96 ± 11.96	141.49 ± 11.68	141.59 ± 12.30	141.62 ± 12.46	0.06
Dietary iron^†^	0.05 ± 0.97	0.01 ± 0.99	0.00 ± 1.00	−0.06 ± 1.03	−0.00 ± 1.00	<0.001
Dietary folate^†^	0.09 ± 0.96	0.04 ± 0.97	0.01 ± 1.00	−0.13 ± 1.04	0.00 ± 1.00	<0.001
Dietary vitamin B12^†^	−0.07 ± 0.97	−0.01 ± 1.00	0.00 ± 1.00	0.08 ± 1.01	0.00 ± 1.00	<0.001
Apo A1 (mg/dL)	153.06 ± 26.85	155.75 ± 27.50	157.52 ± 27.48	160.66 ± 30.04	156.76 ± 28.14	<0.001
Apo B (mg/dL)	108.65 ± 25.90	107.64 ± 26.04	106.88 ± 26.48	105.15 ± 25.68	107.08 ± 26.06	<0.001

Values are expressed as mean ± SD or n (%), unless otherwise indicated.

Values expressed as median (25%, 75%) (for not normal distributed).

Analysis of variance or Pearson Chi-Square was used to calculate p-value for the association across RDW quartiles.

^†^Dietary intake of iron, folic acid and vitamin B12 is adjusted for total energy intake and expressed as standardized residuals.

### All-cause mortality in relation to RDW

A total of 9388 individuals (4715 men and 4673 women) died during 19.8 ± 5.5 (means ± SD) follow up years. The mortality rate was 17.48 per 1000 person-years. Subjects in the fourth versus first quartile of RDW had a significantly higher risk for mortality (HR = 2.02, 95%CI: 1.91–2.15) in model 1 (Table 2). The risk for mortality remained significant adjusted for confounding factors in model 3 (HR = 1.34, 95%CI: 1.24–1.45) (Table 2). The risk for all-cause mortality per 1 SD increment of RDW was similarly significant after adjustment for possible confounders (HR = 1.12, 95%CI: 1.09–1.14).

**Table 2:** Red cell distribution width (RDW) in relation to all-cause mortality.

Sex-specific quartiles of RDW	Q1 (low)	Q2	Q3	Q4 (high)	P value (Q4 vs Q1)
N of death	1767	2117	2428	3076	
HR(95%CI), Model 1^a^	1	1.24(1.17–1.32)	1.49(1.40–1.58)	2.02(1.91–2.15)	<0.001
HR(95%CI), Model 2^b^	1	1.10(1.03–1.17)	1.22(1.15–1.30)	1.57(1.48–1.67)	<0.001
HR(95%CI), Model 3^c^	1	1.08(1.01–1.15)	1.16(1.08–1.24)	1.34(1.24–1.45)	<0.001

^a^Crude model;

^b^Adjusted for age and sex;

^c^Adjusted for age, sex, BMI, current and occasional smokers, use of BP lowering drugs, anaemia, diabetes, MCV, systolic BP, use of lipid lowering drugs, Apo A1, Apo B, high alcohol consumption, B12, iron, folate, WBC count, low physical activity, low education.

There was a significant interaction between RDW and BMI (HR = 0.95, 95%CI: 0.92–0.99) with respect to all-cause mortality. Therefore, participants were categorised according to BMI (<25 and ≥ 25 kg/m^2^). The HR for mortality for individuals with BMI < 25 kg/m^2^ (n = 12791, deaths = 3903) was Q1 (reference), Q2: HR = 1.18, 95%CI: 1.06–1.31; Q3: HR = 1.20, 95%CI : 1.07–1.34 and Q4: HR = 1.35, 95%CI: 1.20–1.53, respectively. The HR for those with BMI ≥25 kg/m^2^ (n = 14272, deaths = 5485) was Q1 (reference), Q2: HR = 1.03, 95%CI: 0.95–1.12; Q3: HR = 1.15, 95%CI: 1.06–1.25 and Q4: HR = 1.32, 95%CI: 1.20–1.46, respectively.

Since smoking is a potential confounder, we also performed an analysis restricted to never smokers. The association between RDW (4^th^ vs. 1^st^ quartile) and all-cause mortality (deaths = 3071, total = 10259, HR = 1.27, 95%CI: 1.12–1.45) and CVD mortality (deaths = 1009, total = 10259, HR = 1.52, 95%CI: 1.21–1.92) remained significant in analysis of never smokers only.

The C-statistics for all-cause mortality from a model including age and sex resulted in a C-statistic of 0.732 (95%CI: 0.727–0.737) and the estimate increased to 0.737 (95%CI: 0.732–0.742) when RDW was added to the model (increment: 0.005 (95%CI: 0.004–0.006)) (p < 0.001).

### RDW in relation to cause-specific mortality

CVD and cancer were the most common causes of death. RDW was significantly associated with cancer mortality (HR = 1.27, 95%CI: 1.12–1.44), CVD mortality (HR = 1.39, 95%CI: 1.21–1.59), and respiratory disease mortality (HR = 1.47, 95%CI: 1.06–2.03) for 4^th^ vs. 1^st^ quartile (Table 3). The corresponding HR for ischemic heart disease (IHD) mortality (deaths: 1356) was 1.44, 95%CI: 1.18–1.77. For cancer subtypes, RDW was significantly associated with mortality from lung cancer (671 deaths, HR (4^th^ vs. 1^st^) = 1.35, 95%CI: 1.00–1.82, and gastrointestinal cancer (993 deaths, HR (4^th^ vs. 1^st^) = 1.31, 95%CI: 1.04–1.65). However, there was no significant associations between RDW and leukaemia mortality (114 deaths, HR (4^th^ vs. 1^st^) = 1.06, 95%CI: 0.55–2.04), breast cancer mortality among women (210 deaths, 4^th^ vs. 1^st^): HR = 0.83, 95% CI: 0.51–1.35, or prostate cancer mortality among men (297 deaths, 4^th^ vs. 1^st^): HR = 0.95, 95%CI: 0.62–1.45.

**Table 3:** Cause-specific mortality in relation to red cell distribution width.

Cause of death	N of death	HR^a^ 95%CI (Q4 vs Q1)	P^a^ value (Q4 vs Q1)	HR^b^ 95%CI (Q4 vs Q1)	P^b^ value (Q4 vs Q1)
Cancer	3458	1.56(1.41–1.71)	<0.001	1.27(1.12–1.44)	<0.001
CVD	3080	1.42(1.28–1.58)	<0.001	1.39(1.21–1.59)	<0.001
IHD	1356	1.47 (1.25–1.72)	<0.001	1.44 (1.18–1.77)	<0.001
Respiratory diseases	598	3.41(2.62–4.43)	<0.001	1.47(1.06–2.03)	0.02
Neurological diseases	434	1.39(1.04–1.85)	0.03	1.44(1.00–2.09)	0.05
Psychiatric diseases	415	1.45(1.10–1.93)	0.01	1.36(0.94–1.96)	0.10
Injuries and poison diseases	300	1.61(1.17–2.20)	0.003	1.25(0.83–1.88)	0.29
Digestive diseases	254	2.67(1.84–3.88)	<0.001	1.56(0.98–2.48)	0.06
Endocrine diseases	241	0.67(0.47–0.95)	0.03	0.95(0.60–1.51)	0.82

^a^Model adjusted for age, sex;

^b^Model adjusted for age, sex, BMI, current and occasional smokers, use of BP lowering drugs, anaemia, diabetes, MCV, systolic BP, use of lipid lowering drugs, Apo A1, Apo B, high alcohol consumption, B12, iron, folate, WBC count, low physical activity, low education.

## Discussion

In this population-based cohort study, we found that there were significant positive associations between RDW and all-cause and cause-specific mortality during an average of 20 years of follow-up, even after adjustment for several potential confounders. We also observed that increased RDW was associated with increased mortality of cancer, CVD, and respiratory diseases. RDW elevation significantly and positively predicted total mortality and CVD mortality regardless of smoking status.

Although RDW is routinely measured in hematology, it is seldom considered as an informative clinical biomarker outside of anaemia in routine practice. One recent cohort study of 240,477 healthy volunteers in UK biobank cohort^13^ reported that RDW was associated with all-cause mortality over 9 years follow-up, and also with incidence of cancer and CVD. In our study, we demonstrated that elevated RDW was associated with all-cause and cause-specific mortality over a longer follow up, on average 20 years. Participants with high RDW were nearly 27% more likely to die from cancer, and 39% more likely to die from CVD compared to the lowest quartile of RDW. With respect to cancer subtypes, we found that increased RDW had a significant association with increased lung cancer mortality, which was in line with findings from studies of cancer patients^14^. There was no significant association between high RDW and leukaemia mortality. This is in contrast to the study from the UK biobank, which speculated that RDW could reflect early activation in bone marrow^13^ and increased production of red cells. It is possible that low statistical power could explain why no such association was found in MDC. RDW was not significantly associated with mortality from prostate cancer among men and breast cancer mortality among women. Our results were also in line with previous studies of RDW and mortality in various groups of patients with cardiovascular disease or cancer^15–17^.

Previous studies have linked RDW to various liver diseases^10^, and adverse prognosis in patients with gastric cancer^18^, and colorectal cancer^8^. In our study, death due to gastrointestinal cancer was significantly associated with RDW. Gastrointestinal hemorrhage is commonly seen in diseases of digestive organs, which potentially could stimulate erythropoietin (EPO) production and provoke anisocytosis^19^. However, the relationship was not completely attenuated after adjustment for anaemia, which might suggest that gastrointestinal bleeding is not the only reason for the increased RDW.

The pathophysiologic mechanisms explaining the association between RDW elevation and adverse outcomes are not well understood. It has been hypothesized that high RDW could reflect inflammation, oxidative stress, anaemia, altered life-span or reduced deformability of the red cells, all of which could potentially increase the mortality risk. It is known that inflammation is associated with reduced production of RBC^20^ and studies of patients with renal failure have reported reduced responsiveness to treatment with EPO in those with low-grade inflammation^21^. This could result in reduced turn-over of the red cells and increased RDW. Low-grade inflammation is a common feature of many diseases, including CVD and cancer. It is conceivable that this mechanism could increase RDW even in studies of apparently healthy individuals. The lifetime of an erythrocyte is approximately 120 days before it is removed from the circulation. However, the erythrocyte life span could vary substantially between individuals^22^. During the life-span, the density of the cell increases and the surface area gradually decreases^23,24^, which then could lead to a heterogeneous population of RBCs and RDW elevation. Hence, increased RDW could be a marker of increased age of the RBCs, possibly due to delayed RBC clearance. Patel *et al*.^25^ proposed that delayed RBC clearance, thereby raised RDW, could be a physiological reaction to stress and poor health. This could potentially explain the relationships between RDW and mortality and the relationships with poor prognosis in many different patient groups. If so, RDW could be a sensitive and non-specific marker of physiological stress and poor well-being. Whether RDW is useful for studying change in general well-being in a population-based setting remains to be explored.

The population-based cohort with large numbers of subjects and events is an important strength of this study. It was possible to control for a large number of potential confounding factors. However, residual confounding is always a possible cause of bias and cannot be completely excluded. RDW in our study was measured as the width (fL) of the erythrocyte distribution curve at a relative height of 20% above the baseline (RDW-SD)^1^ and not as the coefficient of variation (RDW-CV), which has been used in many other studies. In contrast to RDW-CV, RDW-SD is a measure which is independent of MCV, which could be an advantage. In addition, blood samples for analysis of RDW were collected using heparin as anticoagulant, and not using EDTA, which currently is recommended for analysis of blood cell counts. However, even though EDTA could reduce problems with aggregation of platelets and leukocytes, heparin anticoagulation is sufficient for analyzing red cells^26^. Furthermore, the same laboratory procedures were used for all individuals, and any systematic error with respect to survival is very unlikely. One limitation with this study was that RDW was only measured once at baseline examination and there is a possibility that RDW changed during the follow-up period. However, random variations in RDW^27^ during the follow-up would affect the relationship towards null. Another possible limitation could be misclassification of outcome. Information about all-cause mortality in the Swedish Cause of death register should be accurate and complete, but there is likely some misclassification of cause-specific deaths. A study from north of Sweden^28^ reported high validity (87%) of CVD mortality when CVD was coded on chapter level, and lower validity (55%) when CVD was coded on 4-digit level. Another study^29^ reported high validity for deaths due to malignant diseases (92%) and IHD (87%), while lower validity was reported for death due to COPD (52%). However, it is unlikely that misclassification of cause of death is systematically related to RDW. We also used classification of cause-specific mortality on chapter level^28^ instead of 4-digit level, which should reduce the degree of misclassification.

In conclusion, RDW was associated with all-cause mortality and death from cancer, cardiovascular-, and respiratory diseases, respectively. We propose that high RDW is a significant, but non-specific marker of mortality risk in the general population. The possible mechanism underlying the association between RDW and mortality needs further investigation.

## Methods

### Study population

**Figure 1:**
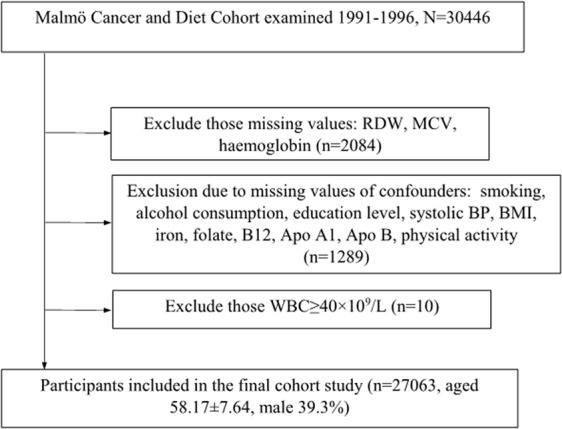
Study population flow chart.

The information used in this study was collected from the Malmö diet and cancer cohort (MDC). Between 1991 and 1996, all men aged 46–73 years and all women between ages 45–73 in the city of Malmö, Sweden, were invited to participate in the study by mail and via newspaper advertisements^30^. A total of 30,446 individuals (12,120 men and 18,326 women) participated in the screening examination, out of an eligible population of 74,138. After exclusion of individuals with missing information on different confounders, the study sample consisted of 27,063 individuals (10,626 men and 16,437 women) with a mean (±SD) age of 58.17 ± 7.64 years (Fig. 1).

### Baseline examination

Venous blood samples were drawn at the first visit at the screening centre. Hemoglobin, white blood cell (WBC) count, and erythrocyte diameter were analyzed in fresh, heparinized blood, using a fully automated assay (SYSMEX K1000 hematology analyzer; TOA Medical Electronics, Kobe, Japan). RDW was calculated as the width (fL) of the erythrocyte distribution curve at the relative height of 20% above the baseline^1^. Reference values were 36.4–46.3 fL in women and 35.1–43.9 fL in men^31^. Anaemia was defined as haemoglobin levels <120 g/L in women and <130 g/L in men (from WHO definition).

Weight and height were measured in light indoor clothing, without shoes. Body mass index (BMI) was calculated as weight/height^2^ (kg/m^2^). Blood pressure was measured using a mercury-column sphygmomanometer after 10 minutes in supine position.

Physical activity, medications, smoking and education level were obtained from the self-administered questionnaire. Smokers were categorized in two categories- smokers (current or occasional smokers) and non-smokers (never smokers or former smokers). Educational level was defined into three categories: school years <9 (low education), 9–12 (medium education) and >12 (high education), respectively^32^. Low physical activity was defined as the lowest quartile of a physical activity score. The physical activity score was based on a self-administrated questionnaire, with questions about different physical activities during different seasons. The physical activity score was created by multiplying the number of minutes per week for each specified activity by an intensity coefficient^33^.

Intake of iron, folate, and vitamin B12 was based on an extensive diet assessment. A 168 item questionnaire for assessment of consumption frequencies and portion sizes and a dietary interview was performed. The procedures have been described elsewhere^34^. Iron, folate, and vitamin B12 intakes were log-transformed and standardized for total energy intake, and adjusted for the method of dietary assessment. High alcohol consumption was defined as >40 g alcohol per day for men and >30 g per day for women.

Diabetes was defined as self-reported physician-diagnosed diabetes or current use of diabetes medication^35^.

Information about mortality and cause of death was retrieved from the Swedish cause of death register^36^. This register covers all deaths among Swedish citizens. Death certificates with information about cause of death are written by a registered physician, and is available for >99% of death according to ICD-9 and ICD-10 diagnostic standards, including cancer ICD-9: 140–239, ICD-10: C00-D49, cardiovascular diseases ICD-9: 390–459, ICD-10: I00–99, ischemic heart diseases (IHD) ICD-9: 410–414, ICD-10: I20-I25, respiratory diseases ICD-9: 460–519, ICD-10: J00-J99, neurological diseases ICD-9:320–389, ICD-10: G, H, psychiatric diseases ICD-9: 290–319, ICD-10: F; injuries and poison diseases ICD-9: 800–999, ICD-10: V,W, X, Y; digestive diseases ICD-9: 520–579, ICD-10: K00-K95; and endocrine diseases ICD-9: 240–279, ICD-10: D80-D89, E^36^. For cancer mortality, we also studied subgroups of mortality among breast cancer (ICD-9: 174, ICD-10: C50), prostate cancer (ICD-9: 185, ICD-10: C61), lung cancer (ICD-9:162, ICD-10: C34), gastrointestinal cancer (ICD-9: 150–159, ICD-10: C15-C26); and leukaemia mortality (ICD-9: 204–208, ICD-10: C91-C95) (from ICD9Data.com and ICD10Data.com).

### Statistical analysis

Mean corpuscular volume (MCV) values were base-e logarithmically transformed before analysis, due to skewed distribution. The Pearson chi-square test was used to determine if there was a significant difference between the expected and observed frequencies in one or more categories. ANOVA was used to analyse the differences among group means. The values of RDW were divided into four sex-specific quartiles (Q), and the Q for men and women were collapsed forming four groups with similar number of men and women in each quartile. The first quartile reflect the erythrocytes that vary the least in size and the fourth quartile reflect the erythrocytes that vary the most in size. RDW was tested as a continuous variable as well (per 1 standard deviation (SD), i.e. 3.553 fL). To describe baseline characteristics of participants in each quartile, continuous variables are presented as means ± SD (or medians (25%, 75%) for skewed distributions), and categorical variables are presented as percentages.

**Figure 2:**
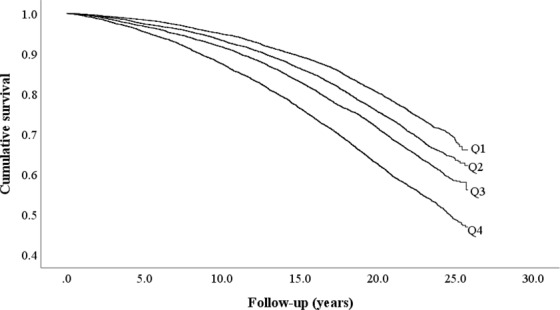
Cumulative survival of all-cause mortality across sex-specific quartiles (Q1–Q4) of baseline red cell distribution width.

The Cox proportional hazards regression was used to examine the association between RDW in different sex-specific quartiles and its association to all-cause mortality with adjustments for risk factors. Hazard ratios (HR) with 95% confidence intervals (CI) were presented. Model 1 was crude model, model 2 was adjusted for age and sex and model 3 was adjusted for risk factors. The Kaplan–Meier curve and log-rank test were used to study all-cause mortality across sex-specific quartiles of RDW (Fig. 2). Potential interactions between exposure variables were tested by interaction terms in the Cox models. The model discrimination was tested with Harrell’s C-statistics^37^ for models using risk factors with and without the RDW and 95% CI were calculated.

IBM SPSS Statistics V.25 (www.spss.com) and STATA version 12 (StataCorp LP, College Station, TX, USA) were used for statistical analyses. P < 0.05 was considered statistically significant.

### Ethical approval and informed consent

All participants volunteered and signed an informed consent. The study complies with the Declaration of Helsinki, and Lund University Ethical Committee (LU51/90 and LU166/2007) approved the study. All data is anonymized both during analytic work and publication, which means no identity will be revealed.

## Data Availability

The authors do not own the data underlying this study. The data are owned by Lund University, and data are available upon request for interested researchers by applying to MDCS steering committee, although restrictions may apply for legal reasons. Please email (anders.dahlin@med.lu.se) with request for the data.
